# Evidence for the Existence of Triple-Negative Variants in the MCF-7 Breast Cancer Cell Population

**DOI:** 10.1155/2014/836769

**Published:** 2014-03-04

**Authors:** Euphemia Leung, Ji Eun Kim, Marjan Askarian-Amiri, Graeme J. Finlay, Bruce C. Baguley

**Affiliations:** Auckland Cancer Society Research Centre, University of Auckland, Auckland, New Zealand

## Abstract

The MCF-7 line, derived in 1973 from a malignant pleural effusion, is one of the most commonly used culture models for human breast cancer. Despite its long history, MCF-7 is a surprisingly heterogeneous line. We previously showed that if MCF-7 cells were cultured for a prolonged period either in the absence of estrogen or in the presence of the antiestrogen tamoxifen, sub-lines were selected that differed from the parental line in ploidy, mean cell volume, signaling pathway usage, and drug sensitivity. This suggests a process of selection of preexisting variants rather than of adaptation of the parental line. All the sublines were estrogen receptor (ER) positive, raising the question of whether MCF-7 also contains ER negative variants. Here, we have looked for such variants by culturing for a prolonged period in the presence of fulvestrant, an estrogen antagonist that has no estrogen agonist activity. Three sublines were developed, each of which was ER negative, progesterone receptor (PR) negative and expressed only a low level of HER2. Each of the variants differed from the original MCF-7 line in ploidy, modal cell volume, and signaling pathway usage. Control experiments in which cells were cultured for a prolonged period in the absence of estrogen selected for variants that were ER and PR positive. The properties of the triple-negative MCF-7 were compared with those of an existing triple-negative cell line, MDA-MB-231, and human epidermal growth factor receptor 2 (HER2)+ SKBr3, as well as from those of the “immortalized” breast epithelial line MCF10A. The results suggest that new variants or phenotypes of MCF-7 might be generated continuously in culture, and by implication this might apply to breast cancer development and even normal breast epithelial development *in vivo*.

## 1. Introduction

Breast cancer is the most commonly diagnosed malignancy in women. Approximately 10% of breast cancers are ER/PR+ HER2+, 69% are ER/PR+ HER2−, 7% are ER/PR− HER2+, and the remaining 13% are classified as triple-negative (TN) [1]. The majority of breast cancers are ER+ in postmenopausal women [2] and low levels of circulating estrogen appear to stimulate breast cancer cell proliferation [3]. Tamoxifen, a selective ER-modulator (SERM) that binds to the ER and prevents the binding of estrogen, has been the standard therapy for ER+ breast cancer. However, tamoxifen also has estrogen agonist activity that may contribute to treatment failure [4] and it has been hypothesized that tamoxifen may promote ER− breast cancer in a subset of women [5]. Fulvestrant (Faslodex) is an ER antagonist that has higher affinity for ER, downregulates ER, and lacks estrogen agonist activity [6]. Development of fulvestrant resistance has also been demonstrated [7]. TN breast cancer is resistant to both tamoxifen and fulvestrant, and the development of effective therapies for TN breast cancer is a focus of current interest [8].

**Figure 1:**
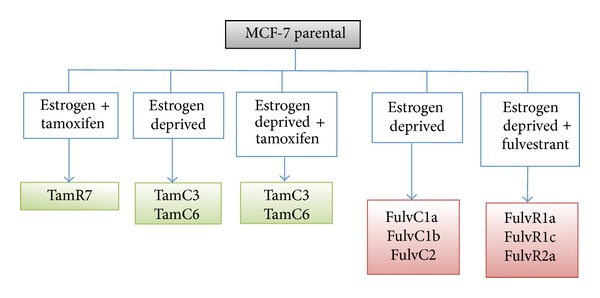
Summary of the MCF-7 parental and sublines, indicating the conditions of subline generation.

Breast cancer is a heterogeneous disease comprised of distinct biological subtypes, and cells with differing receptor status may coexist in the same tumour [9, 10]. Using the ER+ human breast cancer line MCF-7, we have previously sought to develop hormone-resistant sublines by growth in the presence of tamoxifen or in the absence of estrogen [11]. The scheme for the development of these lines is shown in Figure 1 and the resulting sublines demonstrated a range of phenotypes with different drug sensitivity profiles and different receptor status [11–13]. Data for cellular DNA content, modal cell volume, and proliferation rate suggest that the MCF-7 line is heterogeneous and that the sublines grew from minor existing variants rather than by adaptation of the major population. Surprisingly, all the MCF-7 sublines were found to be ER+, despite the measures taken to prevent estrogen signaling. We hypothesize here that either the estrogen agonist activity of tamoxifen or trace amounts of estrogen in the selection medium may have maintained cellular expression of ER receptor and that selection of MCF-7 cell lines using fulvestrant, which lacks ER agonist activity [6], could lead to the emergence of ER negative MCF-7 sublines. In this paper we have extended our original selection strategy [11] to include continuous exposure to fulvestrant (Figure 1) and examined the physical properties, signaling pathways, and drug sensitivity properties of the emerging sublines.

## 2. Material and Methods

### 2.1. Cell Culture

Culture conditions have been described previously [11]; MCF-7, MCF10A, SKBr3, and MDA-MB-231 were purchased from the American Type Culture Collection (ATCC). MDA-MB-231 cells are characterized as triple-negative/basal-B mammary carcinoma and SKBr3 are HER2+ mammary carcinoma. Cells were grown in *α*-MEM containing 5% fetal bovine serum (FBS). All growth media contained insulin/transferrin/selenium supplement, added according to the manufacturer's instructions (Roche), as well as penicillin/streptomycin (100 U/mL and 100 *μ*g/mL, resp.). The FulvR1a, FulvR1c, and FulvR2a cell lines were generated by growth of MCF-7 cells in phenol red-free RPMI containing 5% charcoal-stripped fetal bovine serum (Invitrogen, Auckland, New Zealand), over a period of 3 months in progressively increasing concentrations of fulvestrant (1 nM to 100 nM in ethanol), and then maintaining them for >12 months in 100 nM fulvestrant. The FulvC1a, FulvC1b, and FulvC2 cell lines were generated by exposure of MCF-7 cells for >12 months to the above growth medium but lacking fulvestrant. All experiments were carried out on cells grown in their respective growth media but without fulvestrant.

### 2.2. Verification of Cell Line Identity

The relationship between the derived cell lines and the parental line was established using the PCR Amplification kit which amplifies 15 tetranucleotide repeat loci plus the amelogenin gender-determining marker, performed by DNA diagnostic laboratory (Auckland, New Zealand). The combination of markers selected was consistent with the National Institute of Standards and Technology database recommendations for identity testing.

### 2.3. Modal Cell Volume

Modal cell volume (pl) was determined with the Coulter Counter analysis function.

### 2.4. Chemicals and Reagents

Fulvestrant and tamoxifen were purchased from Sigma (Auckland, New Zealand). Everolimus was from Selleck Chemicals (Houston, TX, USA). NVP-BEZ235 [14, 15] was synthesized according to published protocols.

### 2.5. Cell Proliferation Assay

Cell proliferation was measured using a thymidine incorporation assay in which 3000 cells were seeded in 96-well plates in the presence of varying concentrations of inhibitors for 3 days, except for experiments performed for fulvestrant inhibition (6 days). Briefly, 0.04 *μ*Ci of ^3^H-thymidine was added to each well and incubated for 5 h, after which the cells were harvested onto glass fiber filters using an automated TomTec harvester. Filters were incubated with Betaplate Scint and thymidine incorporation counted in Trilux/Betaplate counter. Cell proliferation was determined by the percentage of cells showing incorporation of ^3^H-thymidine into DNA relative to control (non-drug-exposed) cultures.

### 2.6. Western Blotting

Cells were grown to log-phase, washed twice with ice-cold PBS, and lysed in SDS lysis buffer according to the manufacturer's protocol (Cell Signaling Technology, Danvers, MA, USA). Protein concentration was quantified using the BCA protein assay reagent bicinchoninic acid (Sigma). Cell lysates containing 25 *μ*g of protein were separated by SDS-PAGE and transferred to PVDF membranes (Millipore). Membranes were immunoblotted with antibodies against phospho-Akt (S473), total Akt, phospho-p70S6K (T389), total p70S6K, phospho-rpS6 (S235/236), total rpS6, phospho-ERK (T202/Y204), total ERK, (all from Cell Signaling Technology), tubulin (Sigma), CK5 (Leica), and actin (Millipore), using SuperSignal West Pico (Thermo Scientific, Waltham, MA, USA) or ECL advance (GE Healthcare, Auckland, New Zealand) chemiluminescence reagents. Antibody reactivity was visualized using the chemiluminescence detection system by Fujifilm Las-3000.

### 2.7. Statistical Analysis

Statistical analysis was performed using SigmaPlot. Data were analyzed using either one-way ANOVA coupled with multiple comparisons versus treatment control applying the Holm-Sidak method.

## 3. Results

### 3.1. Characterization of MCF-7 Phenotypes and Comparison of the Physical and Growth Properties of the MCF-7 Cell Line and Its Sublines

**Figure 2:**
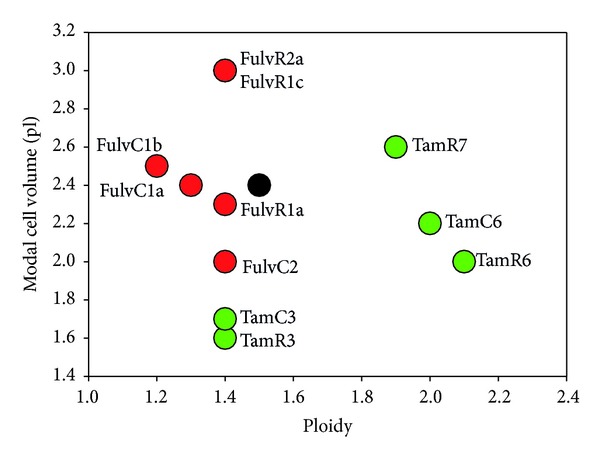
Relationship between DNA content (ploidy) and modal cell volume (pl) for the MCF-7 parental and sublines.

MCF-7 breast cancer sublines were established from the parental line under conditions that mimicked clinical situations involving either prolonged treatment with fulvestrant or estrogen depletion due to oophorectomy or treatment with aromatase inhibitors (Figure 1). MCF-7 cells were cultured either in the presence of increasing concentrations of fulvestrant (top concentration of 100 nM) (FulvR1a, FulvR1c, and FulvR2a) or in the absence of estrogen (FulvC1a, FulvC1b, and FulvC2). Cell lines were characterized by cellular DNA content (ploidy) and modal cell volume. Large differences among individual cell lines were found, allowing them to be separated into five groups. Two sublines, FulvR2a and FulvR1c, selected in the absence of estrogen, produced similar phenotypes, while the rest of the sublines had different properties. A dotplot comparison was made of the DNA content (ploidy) and modalcell volume (pl) of the MCF-7 cell line and its sublines, including the previously developed tamoxifen resistant series of sublines (Figure 2) [11].

### 3.2. Sublines Selected with Fulvestrant Lack ER, PR, HER2, EGFR, and CK5 Expression

**Figure 3:**
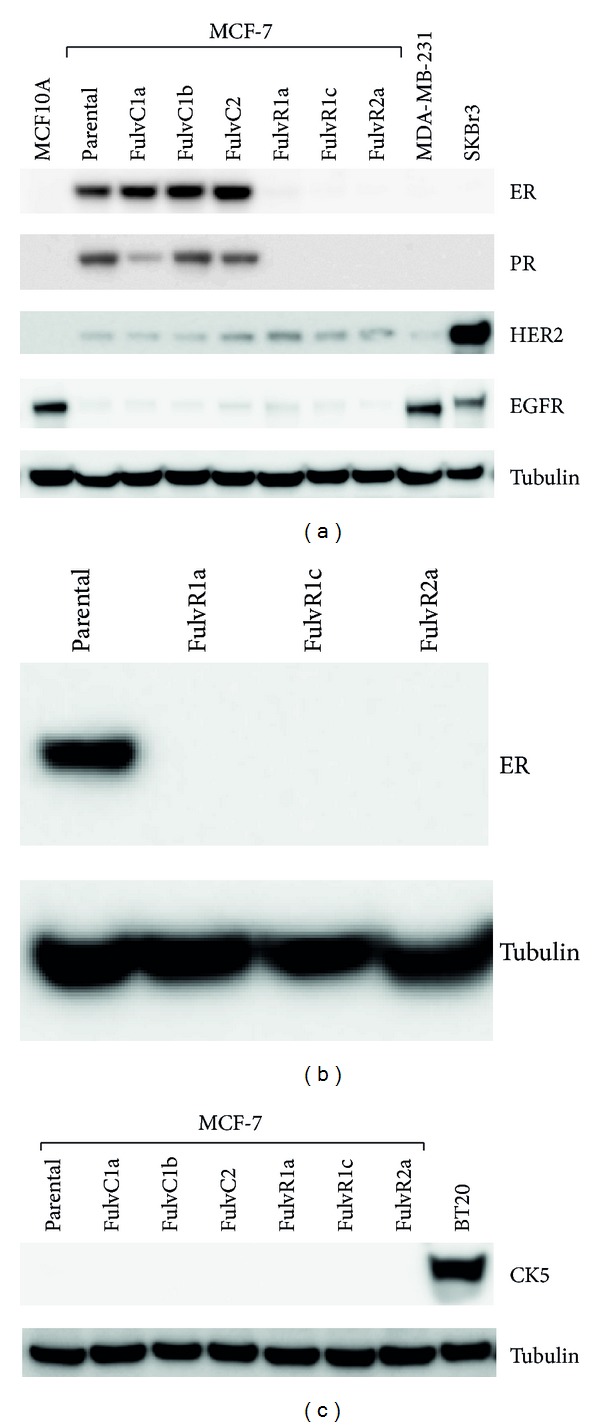
(a) Relative expression of estrogen receptor (ER), progesterone receptor (PR), HER2 and epidermal growth factor (EGFR) in MCF10A (immortalised breast epithelial cells), MCF-7 and its sublines, MDA-MB-231 (triple-negative), and SKBr3 (HER2 positive) breast cancer cell lines. Tubulin is shown as loading control. (b) Relative expression of estrogen receptor (ER) in MCF-7 and its sublines cultured without fulvestrant. (c) Relative expression of CK5 in BT20 (CK5 positive), MCF-7 and its sublines. Tubulin is shown as loading control.

FulvC1a showed reduced PR expression while FulvC1b and FulvC2 had similar receptor levels to those of parental MCF-7 cells. Treatment with the pure antiestrogen fulvestrant led to a downregulation of ER and PR expression in FulvR1a, FulvR1c, and FulvR2a MCF-7 sublines, while no change in HER2 and EGFR expression was observed (Figure 3(a)). To determine whether the ER downregulation was transient, cells were cultured in the absence of fulvestrant for over 2 weeks and ER could not be detected, indicating the fulvestrant treated cell lines had indeed lost ER expression (Figure 3(b)). We examined the cytokeratin 5 (CK5) expression level in the MCF-7 sublines since CK5 has been detected in an ER−/PR− subpopulation of the ER+ T47D breast cancer cell line [16]. However, CK5 was not detected in any of the MCF-7 sublines (Figure 3(c)).

### 3.3. Characterization of AKT/mTOR and ERK Signaling Pathways

**Figure 4:**
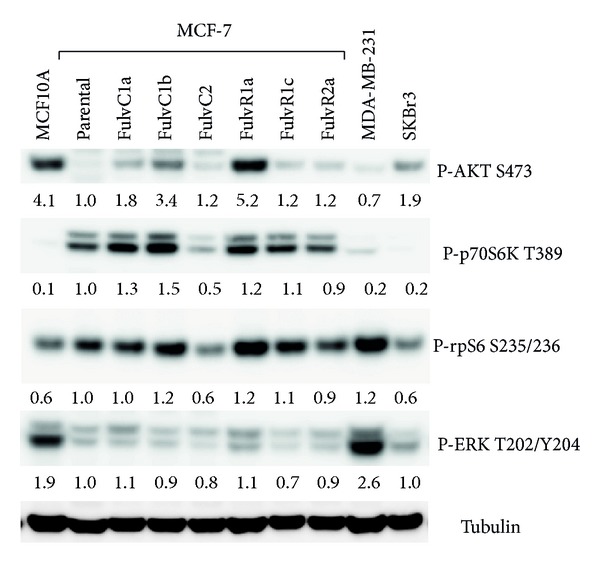
Relative expression of phosphorylated forms P-AKT, P-p70S6K, P-rpS6, and P-ERK. Tubulin is the loading control. Bands are normalised to tubulin control.

The phosphorylation status of Akt, p70S6K, rpS6, and ERK in MCF-7 cells and its sublines was compared with that of normal breast epithelial MCF10A cells, HER2+ SKBR3, and triple-negative MDA-MB-231 (Figure 4). Both the phosphorylated p70S6K and phosphorylated rpS6 expression were reduced in the FulvC2 as compared to the parental and other sublines. The degree of phosphorylation of the other proteins did not differ markedly from that of the parental MCF-7 cells.

### 3.4. Sensitivity of MCF-7 Sublines to Fulvestrant, Everolimus, and NVP-BEZ235

**Figure 5:**
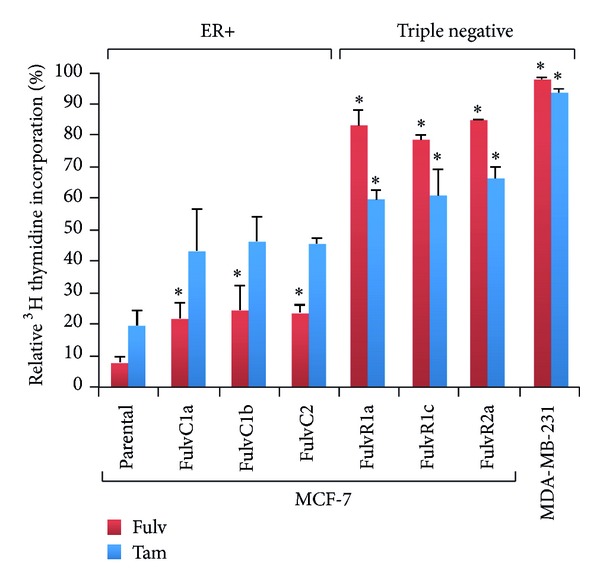
Effects of antiestrogen fulvestrant on the proliferation of MCF-7 parental and derived triple-negative sublines. Data for the triple-negative MDA-MB-231 cell line are shown for comparison. The MCF-7 parental line and its sublines were exposed to fulvestrant (Fulv; 100 nM) or tamoxifen (Tam; 1000 nM) for 6 days and cell proliferation was measured by a thymidine incorporation assay. *Significantly different from MCF-7 parental line (Holm-Sidak test; *P* < 0.05).

A [^3^H]-thymidine incorporation assay was used to assess the effect of drugs on cell proliferation. All MCF-7 sublines showed significant resistance to fulvestrant, with fulvestrant selected sublines showing the strongest resistance relative to the parental cell line (Figure 5). Consequently, even MCF-7 sublines that had been grown in the absence of estrogen but not previously exposed to fulvestrant became less sensitive to fulvestrant. As expected, significant resistance to tamoxifen was observed in the triple-negative fulvestrant treated sublines (Figure 5).

**Figure 6:**
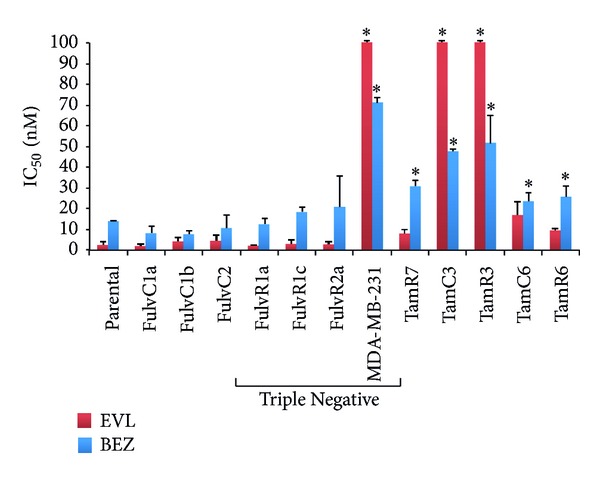
Growth inhibitory concentrations for MCF-7 and its sublines exposed to everolimus (EVL) and NVP-BEZ235 (BEZ). IC50 values (50% inhibition of growth) are shown. The highest drug concentration for everolimus is depicted where 50% growth inhibition was not reached. Cells were treated with drugs for 3 days and cell proliferation was measured by the [^3^H]-thymidine incorporation assay. Bars indicate standard errors in two independent experiments. *Significantly different from MCF-7 parental line (Holm-Sidak test; *P* < 0.05).

The effects of the mTOR inhibitor everolimus [17] and the dual PI3K/mTOR inhibitor NVP-BEZ235 [18] on the proliferation of the MCF-7 parental line and its sublines were determined by [^3^H]-thymidine incorporation assay (Figure 6). Proliferation of MCF-7 and its sublines was inhibited by everolimus (mean IC50 [nM] ± SE for MCF-7, 2.6 ± 1.4; FulvC1a, 2.1 ± 0.8; FulvC1b, 4.2 ± 2.0; FulvC2, 4.5 ± 2.7; FulvR1a, 2.2 ± 0.4; FulvR1c, 3.1 ± 2.1; and FulvR2a, 2.8 ± 0.9). NVP-BEZ235 also efficiently inhibits the proliferation of MCF-7 and its sublines (mean IC50 [nM] ± SE for MCF-7, 14.1 ± 0.3; FulvC1a, 8.2 ± 3.5; FulvC1b, 7.7 ± 1.9; FulvC2, 10.7 ± 6.1; FulvR1a, 12.5 ± 2.8; FulvR1c, 18.5 ± 2.3; and FulvR2a, 21.0 ± 14.8).

The drug sensitivity of the triple-negative breast cancer cell line MDA-MB-231 was also determined for comparison. The IC50 was >100 nM for everolimus and 71 nM for NVP-BEZ235, indicating a much higher level of resistance as compared to the MCF-7 triple-negative sublines.

## 4. Discussion

We have previously developed ER+ hormone-resistant sublines of the ER+ human breast cancer line MCF-7 by growth in the presence of tamoxifen or in the absence of estrogen [11]. Those sublines generally showed reduced sensitivity to mTOR and PI3K inhibitors [11–13]. Here, we have demonstrated the isolation of three ER−, PR−, and HER2− (triple-negative) sublines from the ER+ MCF-7 cell line. Selection was made using the antiestrogen fulvestrant, and each subline was found to differ from the parental MCF-7 line in DNA content (ploidy) and mean cell volume, suggesting that it arose from outgrowth of existing minor variants of the parental MCF-7 cells rather than adaptation of the parental line. Microsatellite analysis of the sublines and the MCF-7 parental line has confirmed that all lines tested are closely related. A surprising feature of the results is that the control cell lines for tamoxifen and fulvestrant were separately obtained by subculturing in estrogen-deprived medium but showed different ploidy and modal cell volume. However, we have previously observed this phenomenon in which two independent cultures grown under the same conditions can lead to the outgrowth of divergent phenotypes [11]. A possible explanation is that cells grow cooperatively in long-term culture and that random cell-cell interactions in minor surviving populations can lead to selection and emergence of sub-populations with different ploidy and other properties, including drug sensitivity. Our data extend findings reported by others that fulvestrant treatment of MCF-7 cells for 18 months was selected for an ER− phenotype [19] while treatment for a shorter duration (21 days) was selected for an ER+ phenotype [20].

Triple-negative breast cancer (TNBC) encompasses an extremely heterogeneous group of tumors [21]. Although the triple-negative subtype is commonly used in breast cancer classification, increasing evidence shows that “basal-like” and “triple-negative” are not synonymous [22]. TNBC expressing basal markers exhibits shorter disease-free survival than those that do not [21]. More than half of ER+ PR+ breast tumors also contain an ER− PR− CK5+ luminobasal subpopulation exceeding 1% of cells [16]. The rare ER− PR− CK5+ progenitor cells appear to escape endocrine therapy and survive to repopulate the tumor [23]. Rare MCF-7 cells that are double positive for luminal and basal markers have been reported [23], but we were unable to detect expression of CK5 or upregulation of EGFR as basal markers in the MCF-7 sublines. An ER− PR− subpopulation has also been selected from the ER+ PR+ T47D breast cancer cell line from orthotopic solid tumors in immune compromised mice [16]. Another study indicated that luminal breast cancer lines contain subpopulations of CK5+ cells that were ER− and resistant to therapy [24]. It is of interest that exome sequencing of circulating tumor DNA in plasma of breast cancer patients indicated the presence of genetic variants that were present as minor populations but could emerge following extended drug treatment [25]. Each of these observations supports the concept of intratumoral heterogeneity, which has now been well documented in breast cancer [9, 10].

The emergence of triple-negative breast cancer in response to treatment is a serious clinical problem and the choice of appropriate therapy is an important consideration [26]. There is currently considerable interest in the use of mTOR inhibitors in the treatment of breast cancer in general [17], and everolimus has been considered as a candidate drug for triple-negative breast cancer [26]. Activating mutations in *PIK3CA* are frequent in breast cancer [27] and inhibitors of PI3K have also been suggested for triple-negative breast cancer [28]. Surprisingly, all the MCF-7 sublines generated in this study showed similar sensitivity profiles to the mTOR inhibitor everolimus and the PI3K/mTOR inhibitor NVP-BEZ235, regardless of the cell culture conditions (i.e., estrogen-deprived, with or without fulvestrant). It has been hypothesized that epidermal growth factor receptor and CK5/6 are positive predictive markers of the triple-negative breast cancer (TNBC) response to everolimus [26], although our data do not support this hypothesis.

TNBCs as a subtype show a more heterogeneous transcriptome and varied histological features [29, 30]. They are often associated with basal markers, are hormone independent, and require aggressive chemotherapy. However, not all patients with TNBC and residual disease after neoadjuvant chemotherapy have worse survival than those with luminal subtypes [31]. The results of the current study, together with other reported data, suggest not only that human breast cancer is heterogeneous, but also that it generates multiple phenotypes that cross the traditional boundaries such as basal and luminal characteristics, that are traditionally used to classify this malignancy.

## Abbreviations

ER+: Estrogen receptor positive
PR: Progesterone receptor
mTOR: Mammalian target of rapamycin
PI3K: Phosphoinositide-3-kinase
*α*-MEM: Alpha-Modified Eagle's Medium
PBS: Phosphate buffered saline
FBS: Fetal bovine serum
rpS6: Ribosomal protein S6
SDS-PAGE: SDS-polyacrylamide gel electrophoresis
PI: Propidium iodide
EGFR: Epidermal growth factor receptor 1
HER2: Human epidermal growth factor receptor 2 (also known as ErbB-2, ERBB2)
Erk: Extracellular signal-regulated kinase
TNBC: Triple-negative breast cancer
CK5: Cytokeratin 5.
